# Wet-spinning fabrication of shear-patterned alginate hydrogel microfibers and the guidance of cell alignment

**DOI:** 10.1093/rb/rbx017

**Published:** 2017-06-30

**Authors:** You Yang, Jing Sun, Xiaolu Liu, Zhenzhen Guo, Yunhu He, Dan Wei, Meiling Zhong, Likun Guo, Hongsong Fan, Xingdong Zhang

**Affiliations:** 1 National Engineering Research Center for Biomaterials, Sichuan University, Sichuan, Chengdu 610064, P. R. China; 2 Department of Gastroenterology, Hospital of the University of Electronic Science and Technology of China and Sichuan Provincial People's Hospital, Sichuan, Chengdu 610072, P. R. China

**Keywords:** tissue engineering, wet-spinning, cell-matrix alignment, multi-hierarchical scaffold

## Abstract

Native tissue is naturally comprised of highly-ordered cell-matrix assemblies in a multi-hierarchical way, and the nano/submicron alignment of fibrous matrix is found to be significant in supporting cellular functionalization. In this study, a self-designed wet-spinning device appended with a rotary receiving pool was used to continuously produce shear-patterned hydrogel microfibers with aligned submicron topography. The process that the flow-induced shear force reshapes the surface of hydrogel fiber into aligned submicron topography was systematically analysed. Afterwards, the effect of fiber topography on cellular longitudinal spread and elongation was investigated by culturing rat neuron-like PC12 cells and human osteosarcoma MG63 cells with the spun hydrogel microfibers, respectively. The results suggested that the stronger shear flow force would lead to more distinct aligned submicron topography on fiber surface, which could induce cell orientation along with fiber axis and therefore form the cell-matrix dual-alignment. Finally, a multi-hierarchical tissue-like structure constructed by dual-oriented cell-matrix assemblies was fabricated based on this wet-spinning method. This work is believed to be a potentially novel biofabrication scheme for bottom-up constructing of engineered linear tissue, such as nerve bundle, cortical bone, muscle and hepatic cord.

## Introduction

The hierarchical structures of the human body include various types of fiber-shaped 3 D cellular construct such as blood vessels, neural pathways and muscle fibers [1]. For these native linear tissues, the performance of their specific biofunction essentially depends on the multi-hierarchical characteristics: Local micro- and nanoscale orientation and topographic pattern of the matrix provide potential cues for cell signalling, adhesion, growth and differentiation, to constitute the functional micro-units, which further assemble in aligned way to form functionalized tissue with special mechanical and biological properties in macro scale. As such, the multi-hierarchically oriented characteristic of fibrous matrix, especially in nano scale or submicron scale, play an important role in the oriented spreading and functionalized expressing of cells such as neuron [2], fibroblast [3], myoblast [4]. And the simulation of multi-hierarchically aligned matrix structure as well as the cellular oriented organization is expected to realize the biomimicking of linear tissues from microstructure to functionalization [5, 6]. Peripheral nerve bundle, for example, is regarded as a typical linear tissue comprised by multi-hierarchically fibrous cell-matrix: the nano/submicron-scaled fibrous matrix (proteins and polysaccharides) recruits nerve cells (Schwann cells and neurons) to form micro-scaled nerve fibers (myelinated nerve fiber), and then assembles into macro nerve tissue (nerve bundles) [7]. Here, each myelinated nerve fiber plays a role of conducting nervous impulse as an independent functional unit at micro scale, which further assembles in a highly-ordered form to integrate functionality in macro scale and achieve the function of bearing mechanical motion, sensing environment stimulations, and controlling the target muscles. Obviously, the generation of highly organized fiber bundles with nano/submicro-oriented microstructure, namely the multi-hierarchical characteristic, is important for the bionic construction of nerve tissue [8, 9].

Wet-spinning technique is widely used as a classical method of preparing fiber-based matrix in tissue engineering [10]. In contrast with electro-spinning [11] and melt-spinning [12], wet-spinning technique has many advantages such as low cost, mild condition and high yield. Meanwhile, the diameter of the wet-spinning microfibers can be easily adjusted by controlling the spinning parameters (prepolymer composition, perfusion rate, spinneret diameter, etc.), and the spun fibers are easy to be assembled by further weaving or 3 D-printing to fabricate scaffold with oriented fibrous structure in macro or meso scale [5, 13]. However, it is still difficult for conventional wet-spinning to obtain hydrogel microfibers with nano- or submicron-scaled topography, and thus can hardly realize the fabrication of multi-hierarchically aligned scaffolds from nano/submicron scale to macro scale by one step.

**Figure 1:**
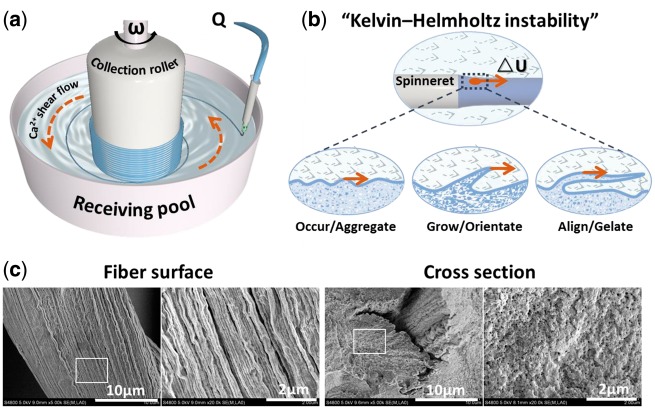
The preparing of shear-patterned hydrogel microfiber based on principle of “kelvin–helmholtz instability”. (**a**) schematic graph of receiving pool in wet-spinning device; (**b**) the evolution process of “kelvin–helmholtz instability waves” on surface of alginate flow; (b) the SEM images of spun microfiber with aligned submicron topography

In this study, a rotary receiving pool is appended to the traditional wet-spinning processes to fabricate hydrogel microfibers with submicron topography, and automatically assemble to obtain the multi-hierarchically aligned fibrous matrix in macro scale. The design is inspired by the “Kelvin–Helmholtz instability”, a transition stage between laminar flow and turbulent flow, which happens when fluid suffering from a shear force in hydromechanics [14]. Here, the rotary receiving pool works in two aspects. Firstly, it mobilizes the circular flow of coagulation solution and exerts a shear force to reshape the surface of hydrogel microfiber with aligned submicron topography. Secondly, under the traction of centripetal force, the spinning hydrogel fibers would continuously twine around the collection roller to form multi-hierarchically aligned fibrous scaffold in macro scale. Specially, alginate hydrogel crosslinked by Ca^2+^ ions was used as material model owing to its property of rapid prototyping [15], in which 2 wt% alginate solution serves as hydrogel prepolymer and 100 mM CaCl_2_ solution serves as coagulation solution. The evolution process of “Kelvin–Helmholtz instability waves” and the resultant aligned submicron topography of alginate hydrogel fibers are shown in Fig. 1. At the outlet of spinneret, the shear flow of Ca^2+^ destroys the laminar conditions on the surface of alginate flow, and leads to the formation of “Kelvin–Helmholtz instability waves”. The instable “waves” undergo an evolution by three stages of “occur/aggregate”, “grow/orientate” and “align/gelate” along with the direction of Ca^2+^ shear flow. Finally, the longitudinally evolutional “waves” on alginate flow surface are timely fixed to form aligned submicron topography owing to the thoroughly gelation of alginate flow. Meanwhile, the spinning hydrogel microfibers can be automatically assembled into aligned fibrous bundle under traction of collection roller.

Furthermore, the contribution of shear force on the orientated submicron topography of spun hydrogel microfibers was systematically investigated by digitally adjusting the rotary rate of receiving pool and perfusion rate of prepolymer. Afterwards, by culturing shear-patterned hydrogel microfibers with PC12 and MG63 cells respectively, we investigated the effect of highly-orientated submicron topography on cell alignment. Finally, dual-aligned fibrous cell-matrix units were observed and further assembled to achieve an *in vitro* mimicking of native multi-hierarchical linear tissues such as nerve bundle. This study provides a new idea for the subsequently “bottom-up” bionic construction of multi-hierarchical linear tissues.

## Materials and methods

### Reagents and apparatuses

Type I collagen (derived from abortive calfskin), Rhodamine-Phalloidin (Thermo Fisher Scientific^TM^, the US), and ﬂuorescent goat anti-rabbit IgG (Invitrogen) were used. Sodium alginate, fluorescein diacetate (FDA) and propidium iodide (PI) were purchased from Sigma-Aldrich^TM^, US. Unless otherwise specified, other chemicals were all provided by Chengdu Kelong Reagent Co., Ltd. Syringe pumps TS2-60 were purchased from Lange Baoding Co. Ltd. (Hebei, China). D2015W electric motor was provided by Shanghai Meiyingpu Instrument Co., Ltd.

### Wet-spinning processes

The spinning process relies on the principle that sodium alginate gelatinizes with Ca^2+^ quickly (the gelation speed is nearly related to the diffusion speed of Ca^2+^) [15]. With the help of self-designed receiving pool in the wet-spinning device shown in Fig. 1a, shear-patterned sodium alginate microfibers can be produced and can be further automatically assembled into highly-aligned fibrous hydrogel mat or bundles with multi-hierarchical structure from micro to macro scale.

The parameters and materials for a typical spinning process are as follows: (1) 2 wt% sodium alginate aqueous solution was prepared as the hydrogel prepolymer, which will be sprayed out from the spinneret driven by the syringe pump, and the perfusion rate Q is set at 10 ml/h; (2) 2/3 of the receiving pool was filled with 100 mM CaCl_2_ aqueous solution to serve as coagulation solution of the hydrogel microfiber here; (3) the collection roller of receiving pool was equipped on an electric motor and the rotation rate ω was set at 80 rpm, which would produce shear force to alginate flow by driving the circling flow of CaCl_2_ aqueous solution. Thus, the hydrogel microfibers can be shear-patterned by the CaCl_2_ shear flow and assembled onto collection roller in a highly-aligned way.

Here, the samples for cell test were prepared and named as following principles. Alginate hydrogel microfibers with aligned submicron topography were denoted as “shear-patterned fiber (SP fiber)”, which was spun at condition of 10 ml/h perfusion rate of alginate and 80 rpm rotation rate of receiving pool; Alginate hydrogel microfibers with non-aligned submicron topography was denoted as “simple-extruded fiber (SE fiber)”, which was prepared by simply extruding at condition of 10 ml/h perfusion rate of alginate and 0 rpm rotation rate of receiving pool; And cells cultured on petri dishes was set as blank control group denoted as “petri dish”.

### Cell culture and testing

The differentiated neuron-like PC12 cells of rat and human osteosarcoma MG63 cells were both purchased from the Type Culture Collection of the Chinese Academy of Sciences, Shanghai, China. Both cells were cultured in a similar way. Briefly, the cells were cultured in DMEM medium supplemented with 10% FBS, 2 mM glutamine, 10 U/ml penicillin and 10 mg/ml streptomycin come from HyClone™ and maintained in standard conditions (37 °C, 95% humidity, 5% CO_2_). Cells were passaged at 80% confluence.

To preserve the sub-micron topography when conducting sterilization to fibrous samples, the alginate hydrogel microfibers were immersed in 30%, 50%, 75% ethanol successively, maintained 15 min for gradient. Then they were immersed in sterile 1 mg/ml type I collagen aqueous solution (pH 6–7) at 4 °C for 8 h to improve surface cellular adherence. PC12 or MG63 cells at 80% confluence were harvested to make a cell suspensions of 2 × 10^5^ cells/ml, and then were pipetted into each well of a 6-well plate, where the sterilized alginate hydrogel microfibers were placed in advance. Cells cultured on a blank well were set as the blank control group. Samples were cultured continuously and the medium was refreshed every 2 days.

### Scanning electron microscope (SEM) imaging

For SEM analysis, samples were successively dehydrated by immersing with 30%, 50%, 70%, 80%, 90%, 95%,100% gradient ethanol, 15 min for each step. Then, samples were critical point dried, sputter-coated with 8-nm thick Au/Pd, and imaged by a field emission scanning electron microscope (FESEM, S-4800, Hitachi, Japan). Notably, samples seeded with cells would be firstly fixed with 2.5% glutaraldehyde before above treating for SEM imaging.

### Cellular fluorescent staining and imaging

Cell surviving and spreading on microfibers were observed by FDA/PI double staining. Briefly, samples were incubated in a mixture of 100 µM FDA and 60 µM PI at room temperature for 30 s and visualized with a confocal laser scanning microscope (CLSM, Leica-TCS-SP5). For observation of cellular cytoskeleton and nuclei, F-actin/DAPI counter staining was applied. Simply, samples were incubated in 6.6 µM Rhodamine-Phalloidin solution at room temperature for 30 min and in 0.1% (v/v) DAPI solution for 5 min, respectively, after which the confocal images were captured. For imaging of neuroflament-200 (NF-200), anti-Neurofilament 200 antibody produced in rabbit (sigma, 1:100 diluted goat serum) was used as primary antibody, and samples were rinsed three times in PBS prior to each step. Samples were firstly rinsed by PBS solution and fixed in ice acetone for 15 min, then TritonX-100 (0.2%, v/v) was added to the samples for 5 min for cell permeabilization. This was followed by immersing with goat serum for 30 min at room temperature to block non-specific adsorption, then incubation with the primary antibody solution at 4 °C overnight, and incubation with the ﬂuorescent goat anti-rabbit IgG (Invitrogen, diluted 1:250 in 10% goat serum) for 1 h at room temperature in the dark. Finally, the samples were incubated in DAPI solution for 5 min to stain the nuclei and then were washed for confocal imaging.

## Results and discussion

### Preparation of shear-patterned alginate microfibers

Here, a rotary receiving pool was appended to conventional wet-spinning apparatus to drive flow of coagulation solution, thus provided a shear force to shape the aligned submicron topography on spun microfibers. In the spinning apparatus as shown in Fig. 1a, the perfusion rate of sodium alginate solution Q (ml/h) can be precisely controlled by the syringe pump, which determines the initial velocity of alginate flow (U_1_, which is positively correlate with Q); The rotation rate ω of receiving pool can be precisely controlled by the electric motor, which determines the flow velocity of CaCl_2_ (U_2_, which is positively correlate withω). Here, △ U (△ U = U_2_-U_1_) is used to present the shear strength that the alginate flow suffers from CaCl_2_ flow at the outlet of spinneret. Obviously, when U_2_> U_1_, the alginate flow surface is subjected to positive shear disturbances from the CaCl_2_ shear flow to form the “Kelvin-Helmholtz instability waves” along with the flow direction. Then, the waves start aggregating, growing, and aligning driven by shear force, and finally are fixed into aligned submicron topography by thorough gelation of alginate microfiber (Fig. 1b). As shown in Fig. 1c, aligned submicron topography was obtained on the outer surface of shear-patterned fibers, while the cross-section remained disorderly amorphous, which further confirmed the hypothesis of “Kelvin - Helmholtz instability”. Since the alignment of submicron topography on spun fiber is highly relative to the shear-strength, which is decided by the rotation rate of receiving pool (ω) and perfusion rate of alginate (Q), the correlation between fiber submicron topography and shear-induced “Kelvin-Helmholtz instability” was systematically discussed by adjusting Q and ω respectively.

### Fiber topography adjusted by rotation rate ω

**Figure 2:**
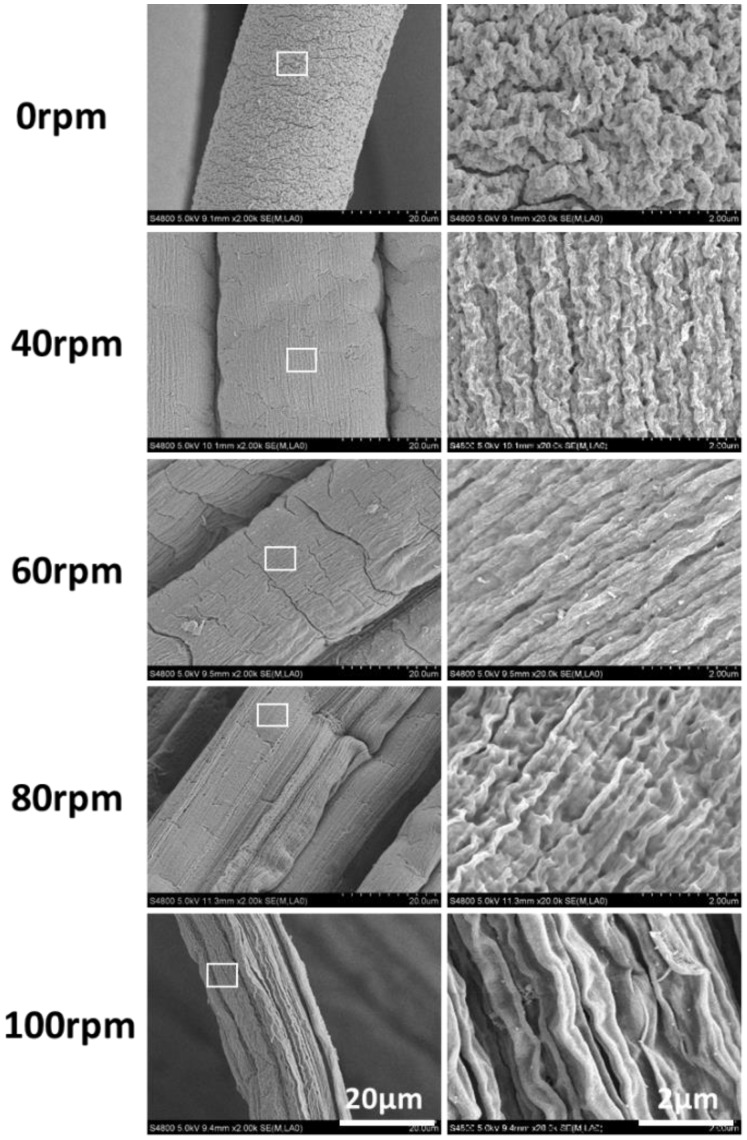
SEM Images of the spun microfiber with different rotation rate ω (rpm) of receiving pool (fixed conditions: 2 wt% sodium alginate, 100 mM of CaCl_2_, Q was fixed at 10 ml/h)

As mentioned above, a higher value of rotation rate ω means a stronger shear strength △U, thus expedites the evolution processes of shear-induced “Kelvin-Helmholtz instability waves”, and finally leads to a more obvious orientation of submicron topography companied with the thorough gelation of alginate flow. When the perfusion rate Q is fixed at 10 ml/h, namely the flow rate (U_1_) of sodium alginate is nearly constant, the shear strength △U (△U = U_2_-U_1_) will increase with the upregulation of rotation rate (ω), and thus leading to the increased orientation trend of submicron topography on hydrogel microfibers. Consequently, the microfiber prepared by simply extruding (0 rpm) presented no obvious aligned submicron topography, while the microfiber spun at 100 rpm occurred with the maximum alignment (Fig. 2).

### Fiber topography adjusted by perfusion rate Q

**Figure 3:**
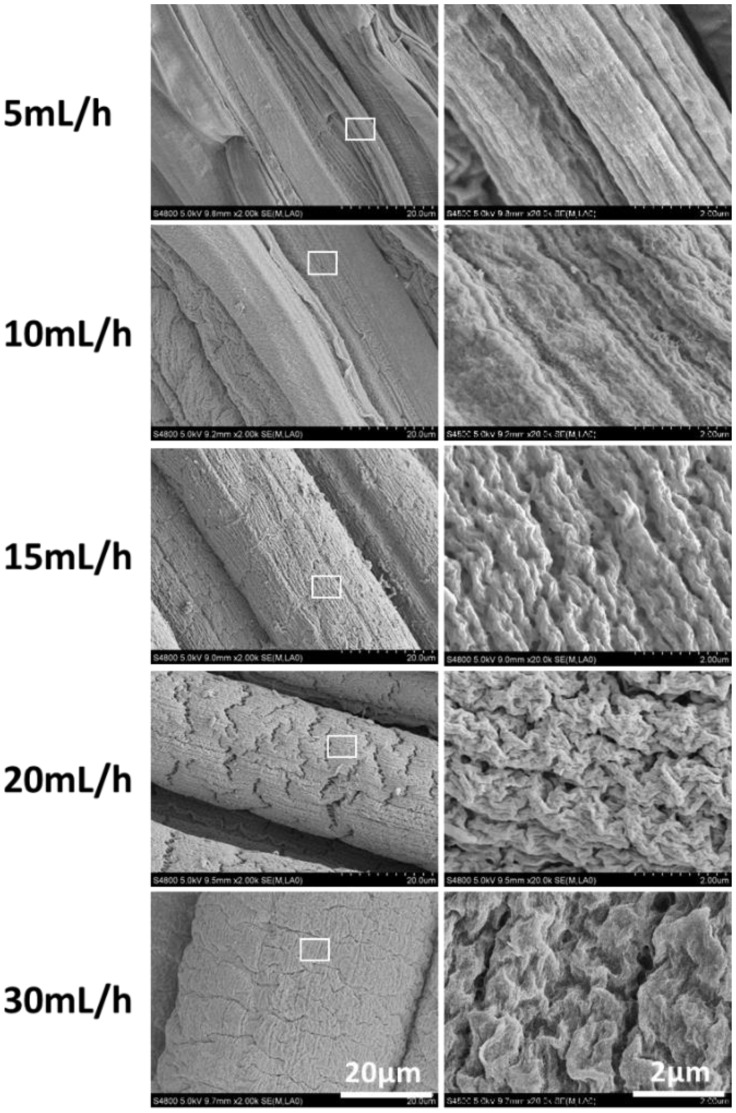
Spun microfiber with different perfusion rate Q (ml/h) of alginate (fixed conditions: 2 wt% sodium alginate, 100 mM of CaCl_2_, ω was fixed at 80 rpm)

Similarly, a lower value of perfusion rate Q would also contribute to a stronger shear strength △U. As is shown in Fig. 3, where the rotation rate of receiving pool ω is fixed at 80 rpm, namely U_2_ is constant, the shear strength △U will decrease with the upregulation of alginate perfusion rate Q, and finally the orientation of the surface microstructure will gradually weaken. Therefore, maximum and minimum alignment of submicron topography happened at the lowest and highest value of Q, 5 and 30 ml/h, respectively.

In general, either a bigger ω or smaller Q can contribute to the increase of shear strength △U, which accelerates the evolution of “Kelvin-Helmholtz instability waves”, thus the “waves” on alginate flow surface can be well aligned as a highly-ordered submicron topography before gelation. Instead, when the value of Q is large but ω is small, meaning the small value of △U, the shear-induced waves can't grow and align thoroughly, and thus result in the weakened aligned topography on the microfiber.

### Orientation of PC12 cells induced by shear-patterned alginate microfiber

Native peripheral nerve bundle is considered as a typical multi-hierarchical tissue that comprises of dual-aligned neurocytes-matrix assemblies [7]. The nano/submicron topography in tissue matrix plays an important role on the function expression of neural cells [16]. Here, the rat neuron-like cell line PC12 was chosen as a cell model to investigate the cell orientation on shear-patterned hydrogel microfibers. Notably, to enhance cell vitality and attachment on alginate fiber, all samples were incubated with 1 mg/L type I collagen solution in prior to cell seeding [17], and the submicron topography of fibers with collagen coated remained impervious (The data is not given here).

**Figure 4:**
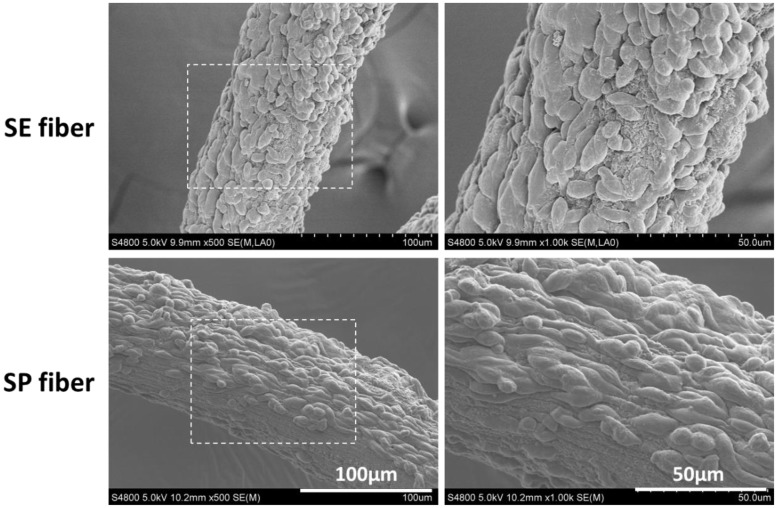
SEM Images of PC12 cells cultured on surface of simple-extruded fiber (SE fiber), and shear-patterned fiber (SP fiber) after 3 days

The attachment and elongation of PC12 cells on microfibers were first observed by SEM images as shown in Fig. 4. On fibers with highly aligned submicron topography (SP fiber), PC12 cells spread and elongated along with the fiber axis. While on SE fiber, the submicron topography is non-directional and the cell orientation also presents in a random distribution. This result shows a consistent tendency with some previous reports [18, 19].

**Figure 5:**
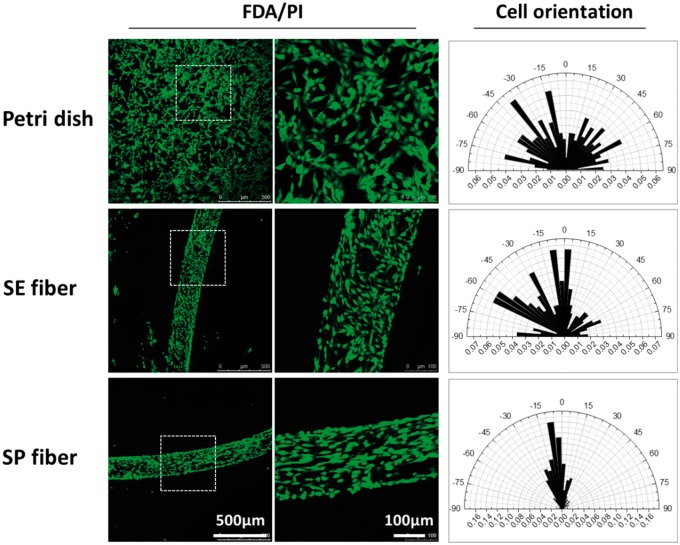
The spreading (FDA/PI staining) and orientation (polar graph) of PC12 cells cultured on the surface of petri dish, simple-extruded fiber (SE fiber), and shear-patterned fiber (SP fiber) after 3 days. The data of cellular orientation was measured on ImageJ 1.5f, national institutes of health, USA, *n* > 200

Afterwards, FDA/PI and DAPI/F-actin staining were used to investigate the viability and morphology of PC12 cells seeding on the hydrogel microfibers. Similarly to the results of SEM, the aligned submicron topography of shear-patterned fiber well induced the alignment of PC12 cells. In Fig. 5, PC12 cells on the surface of SP fiber showed an apparent tendency of orderly spreading along with the fiber axis, while cells on the SE fiber or perti dish showed randomly-oriented spreading and elongation. The orientation tendency of PC12 cells on different interfaces was quantified by the statistical analysis of the cellular orientation angle, which was shown as the polar graphs in the right of Fig. 5. Here, the cell orientation angles were acquired by measuring the intersection angle between cell elongation direction and fiber axis according to the FDA/PI staining images. In particular, only elongated cells with an aspect ratio of more than 2 were counted in the measurement and more than 200 elongated cells were counted for each sample. The results demonstrated that 68.8% of the PC12 cells on SP fiber oriented along fiber axis with angels below 15°, while the orienting ratio for cells on SE fiber and petri dish was only 28.4% and to 13.3%, respectively.

**Figure 6:**
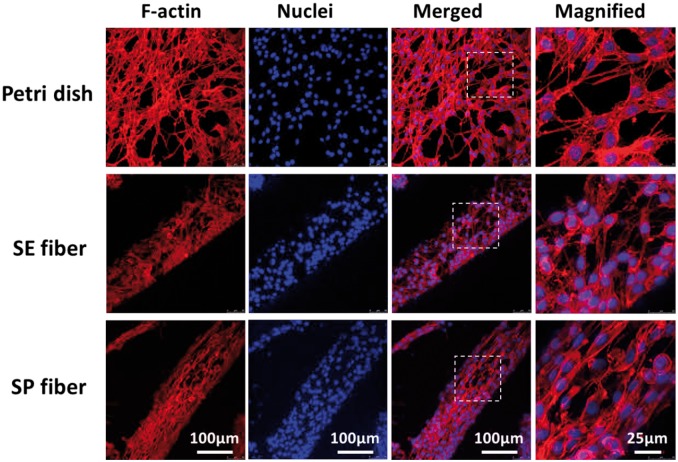
F-Actin/DAPI staining of PC12 cells cultured on surface of petri dish, simple-extruded fiber (SE fiber), and shear-patterned fiber (SP fiber) after 3 days

The alignment of PC12 was further investigated by observing the orientation of cytoskeleton through F-actin/DAPI staining. As an important component of cellular cytoskeleton, F-actin can reshape the cellular morphology and propel cellular motility by reassembling [20, 21], thus dominating cell immigration and elongation, stimulating cell differentiation and functionalization as well [22, 23]. As shown in Fig. 6, cytoskeleton proteins of aligned cells on SP fiber presented an orientated distribution along with fiber axis, and organized stress fibers as well as directional deformation of nuclei can be found in most elongated cells. However, for cells on the surface of SE fiber and petri dish, the orientation of F-actin and nuclei all present a thoroughly random distribution.

**Figure 7:**
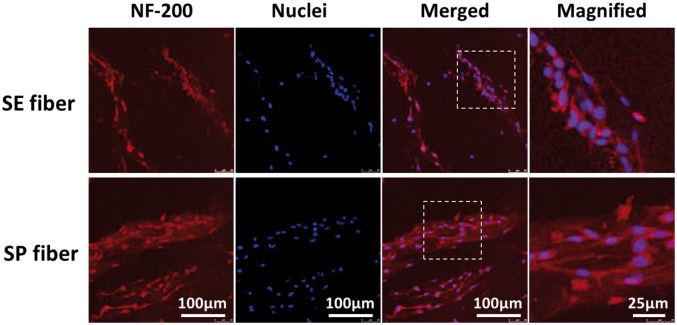
NF-200/DAPI Counter staining of PC12 cells cultured on the surface of simple-extruded fiber (SE fiber) and shear-patterned fiber (SP fiber) after 3 days

Neurofilament (NF), as the main skeleton component of neuronal axon, is a typical marker of mature neuron and plays a vital role in delivering neurotransmitters from cell body to distal synapse [24]. Figure 7 shows the Immunofluorescence staining of anti-neurofilament 200 (NF-200) for PC12 cells cultured on SE fiber and SP fiber after 3 days. Here, an upregulated expression of NF-200 was found on SP fiber compared with that of SE fiber, suggesting that hydrogel fibers with aligned submicron topography could also significantly support the neuron-like differentiation of PC12 cells. In brief, SP fiber could guide PC12 cell to spread and elongate directionally along with fiber axis, and further supports the neuronal phenotype of PC12.

### Orientation of MG63 cells induced by shear-patterned alginate microfiber

**Figure 8:**
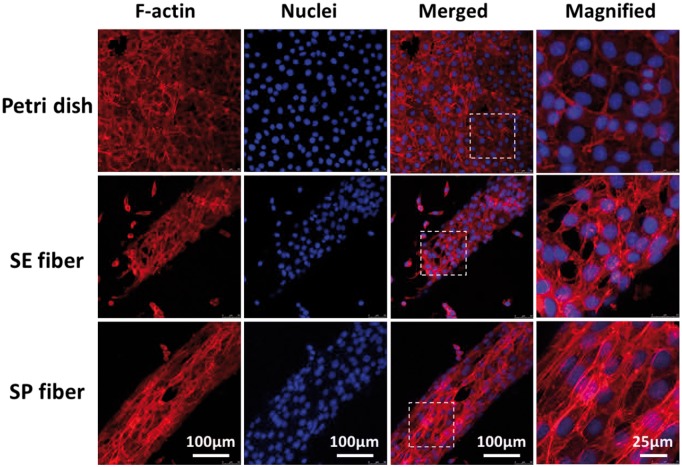
F-Actin/DAPI staining of MG63 cells cultured on the surface of petri dish, simple-extruded fiber (SE fiber), and shear-patterned fiber (SP fiber) after 3 days

Except for peripheral nerve, the multi-hierarchical structures built with highly ordered cell-matrix micro-units are also found in many other native tissues [1]. Cortical bone is another instance, comprising of aligned osteons, which are cylindrical structures stacked by highly organized osteocytes and collagen fibers in micro scale [25]. Human osteosarcoma MG63 cells were recruited to further verify the cell alignment on shear-patterned microfibers. For cells on SP fiber, as shown in Fig. 8, both F-actin and nuclei ware found to distribute with a higher-ordered way along with fiber axis, and F-actin as well as stress fiber expressed at a higher level than that of cells on SE fiber and petri dish. These results indicated that the aligned submicron topography on SP fiber can also effectively support the spreading and orientation of osteocytes [26].

### Construction of multi-hierarchical nerve -like scaffold

**Figure 9:**
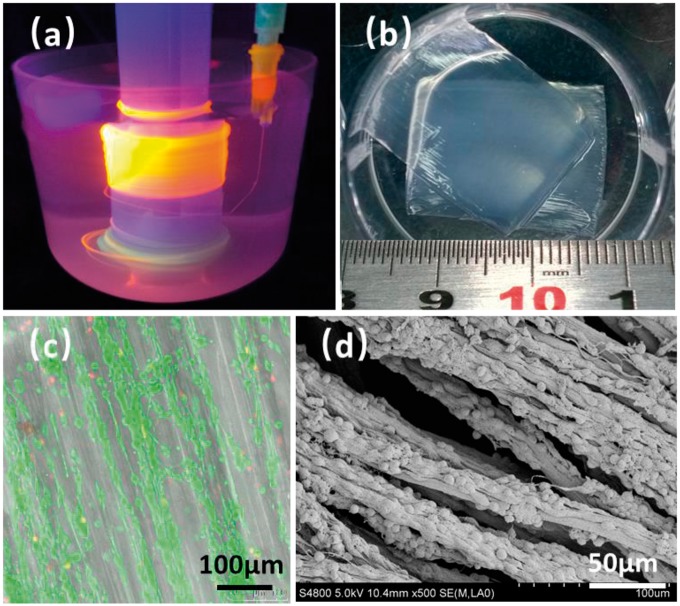
The preparation and characterization of multi-hierarchical nerve-like scaffold. (**a**) wet-spinning and automatically assembling of shear-patterned alginate hydrogel microfiber, in which the hydrogel was colored by orange fluorescent quantum dots, (**b**) the photograph of hydrogel mat prepared by the assembly of alginate microfibers, (**c**) FDA/PI staining and, (**d**) SEM image of PC12 cells co-culturing with the hydrogel mat after 3 days

As is mentioned in introduction, the appending of rotary receiving pool can not only provide a shear-force to shape the submicron topography on spinning microfibers, but also serve as an assembling tool to fabricate aligned fibrous bundle by the rolling of the spun microfibers, finally realizing the building of multi-hierarchically aligned scaffold from micro scale to macro scale. As shown in Fig. 9a and b, shear-patterned microfibers, which can effectively guide cell spreading and elongating directionally, were automatically assembled into hydrogel mat to serve as the multi-hierarchically aligned fibrous matrix of PC12 cells. After 3-dayculture, PC12 cells on the hydrogel mat exhibited good viability and highly ordered spreading along the direction of aligned fibrous matrix (Fig. 9c and d). Although the dual-aligned cell-matrix units can be achieved by many other methods such as photolithography [27], electro-spinning [6], microfluidic technology [28], and 3 D-printing [29, 30], they are often complex, costly and low-yield. By comparison, this improved wet-spinning method here provides a simple way to obtain multi-hierarchically aligned tissue-like scaffold with low cost and high yield.

## Conclusions

The fabrication and automatically assembling of hydrogel microfiber with submicron topography were successfully achieved in one step by appending a rotary receiving pool to the traditional wet-spinning process. Based on the hydromechanical principle of shear-induced “Kelvin–Helmholtz instability”, the aligned submicron topography on shear-patterned fibers could be tuned by controlling the perfusion rate of alginate and rotation rate of receiving pool. *In vitro* culture suggested that these microfibers with orientated submicron topography could guide cell alignment and differentiation effectively. Consequently, dual-aligned fibrous cell-matrix units were observed and further assembled to achieve an *in vitro* mimicking of native multi-hierarchical linear tissues such as nerve bundle. This study provides a new idea for the bionic “bottom-up” constructing of multi-hierarchical linear tissues.

## Author contributions

The manuscript was written through contributions of all authors. All authors have given approval to the final version of the manuscript

## Notes

The authors declare no competing financial interest.

## Funding

This work is supported by the National Natural Science Foundation of China (Contract Grant No. 51473098, and 51673128).

*Conflict of interest statement*. None declared.
